# Outcome and safety of SBRT in centrally and ultra-centrally located lung tumours: A PRISMA-based systematic review and Meta-Analysis

**DOI:** 10.1016/j.ctro.2026.101151

**Published:** 2026-03-21

**Authors:** Shari Wiegreffe, Sonja Adebahr, Tanja Schimek-Jasch, Julian Philipp Layer, Cas Stefaan Dejonckheere, Gustavo Renato Sarria, Davide Scafa, Youness Nour, Lara Caglayan, Dimos Baltas, Christos Moustakis, Nils Henrik Nicolay, Andreas Rimner, Anca-Ligia Grosu, Ursula Nestle, Eleni Gkika

**Affiliations:** a Department of Radiation Oncology, University Hospital Bonn 53127 Bonn, Germany; b Department of Radiation Oncology, Medical Center, University of Freiburg, Faculty of Medicine, German Cancer Consortium (DKTK), partner site DKTK, Freiburg, Germany; c Institute of Experimental Oncology, University Hospital Bonn 53127 Bonn, Germany; d Department of Radiation Oncology, University Hospital Leipzig 04103 Leipzig, Germany; e Department of Radiation Oncology, Kliniken Maria Hilf GmbH Mönchengladbach 41063 Mönchengladbach, Germany

**Keywords:** Stereotactic body radiotherapy, Ultra-centrally, Centrally, NSCLC, Systematic review, Meta-analysis

## Abstract

**Introduction:**

Stereotactic Body Radiation Therapy (SBRT) offers a curative treatment option for patients with early-stage central and ultra-central lung tumours, however can be associated with severe side effects. This review provides a structured quantitative evaluation of outcomes and safety in these critical locations, therefore offering safety-oriented evidence to support individualised treatment planning.

**Methods:**

Pubmed/MEDLINE was searched with the term [“Carcinoma, Non-Small-Cell Lung”[Mesh] AND (“Radiosurgery”[Mesh] OR “SABR” OR “stereotactic ablative radiotherapy” OR “SBRT” OR “stereotactic body radiotherapy”)] according to PRISMA-guidelines. Trials reporting on SBRT in central and ultra-central lung tumours and on outcome parameters were included. A *meta*-analysis of proportions was performed using random-effect models.

**Results:**

57 retrospective and 12 prospective trials were included, encompassing in total 3,672 and 1,509 patients with central and ultra-central lung tumours. The median follow-up was 22.7 (range: 10.6 – 93.6) months for centrally and 21.2 (8.6 – 93.6) months for ultra-centrally located tumours. For ultra-central lesions the median overall survival was 28.0 (12.0 – 64.5) months compared to 39.4 (19.0 – 57.0) months in central lesions with highest efficacy of SBRT with 60 Gy in 8 fractions. Overall, pooled grade 3–5 toxicity was low for both localizations. The most frequently reported fatal complication in ultra-centrally vs. centrally located tumours was haemorrhage with an estimated proportion of 0.48% [range: 0 – 14.89; CI 95% = 0.00 – 1.68] vs. 0.12% [range: 0 – 7.09; CI 95% = 0.00 – 0.46].

**Conclusion:**

SBRT with 60 Gy in 8 fractions provides a feasible and safe curative treatment approach for patients with centrally and ultra-centrally located lung tumours.

## Highlights

• SBRT (60 Gy in 8 fractions) is feasible and safe for (ultra-)central lung tumors.
• Prioritization of sparing the PBT over full PTV coverage is recommended.
• To avoid fatal toxicity, Dmax < 60 Gy (EQD2 (α/β = 3) = 126 Gy) to PBT is recommended.

## Introduction

Non-small cell lung cancer (NSCLC) is the leading cause of cancer-related mortality worldwide[1]. Surgery is considered as standard of care for the management of early-stage disease. Due to the high incidence of cancer-unrelated comorbidities like poor cardiovascular and pulmonary function, many patients are not suitable for surgery. Stereotactic body radiation therapy (SBRT) is a high-precision irradiation technique that achieves very high doses within the tumour with steep dose gradients, while the dose exposure to the normal tissue remains low. It therefore provides a highly effective and low-risk curative option especially for functionally or medically inoperable patients with early-stage disease or those declining surgery [2], [3], [4], [5], [6]. Equally, SBRT of malignant lung lesions has proven to be an effective alternative for patients with oligometastatic disease [7].

As of now, the optimal fractionation and dosing scheme remains debatable, while largely depending on the exact tumour location. Available data indicate the necessity of individual balancing between tumour control and toxicity. To yield favourable therapeutical outcomes in peripheral localized lung tumours, a biologically effective dose (BED_10_) greater than 100 Gy has been suggested in previous studies. Especially for tumours abutting central or mediastinal structures, however, there are several limitations due to anticipated toxicity risks [8].

Previous studies reported an increased risk of grade 5 haemorrhage and pneumonitis for 3-fraction SBRT with 60–66 Gy in centrally located lung tumours, while cases of airway necrosis have also been documented. Thus, the so-called “no-fly-zone” was defined as an area of 2 cm around the proximal bronchial tree (PBT), reflecting the historically observed higher risk of severe and potentially fatal toxicity in this region. This designation did not represent an absolute contraindication to SBRT but rather underscored the need for careful patient selection and treatment planning. [9], [10] Further studies focused on evaluating risk-adapted fractionation schemes to reduce overall toxicity for SBRT within this predefined area. Exemplarily, RTOG-0813 and LungTech showed comparable outcome and toxicity rates for SBRT of centrally located lesions with 50–60 Gy in 5–8 fractions [11], [12]. Due to the lack of prospective data on outcome and toxicity of SBRT of tumours adjacent to central or mediastinal structures, the evaluation of SBRT in these tumours became of major interest in research. As a consequence of increased incidence of grade 5 toxicity, tumours overlapping the central airways were defined as ultra-centrally located and were thus differentiated from centrally located tumours [13], [14]. The existence of different definitions for central and ultra-central lung tumours continues to pose a challenge for assessing the effectiveness and toxicity of SBRT in centrally and ultra-centrally located lesions. While several reviews have addressed either central or ultra-central tumours individually, a structured quantitative analysis integrating outcome and toxicity data across both subgroups remains lacking. Therefore, the aim of this PRISMA-based systematic review and *meta*-analysis is to provide a structured overview and to quantitatively evaluate the latest research results regarding outcome and safety of SBRT in both centrally and ultra-centrally located lung tumours.

## Materials and Methods

### Search Strategy

According to the Preferred Reporting Items for Systematic Reviews and Meta-Analyses (PRISMA) statement [15] we performed a comprehensive literature search of the PubMed/MEDLINE database by one author (S.W.). The Medical Subject Headings (*MeSH-Terms*) ‘*Carcinoma, Non-Small-Cell Lung*’ and ‘*Radiosurgery*’ were chosen and combined with the search terms ‘SABR’, ‘stereotactic ablative radiotherapy’, ‘SBRT’ and ‘stereotactic body radiotherapy’ to screen trials published between October 1, 1997 and February 11, 2025. To extend the literature search, a complementary cross-search of already included articles was performed.

**Fig. 1:**
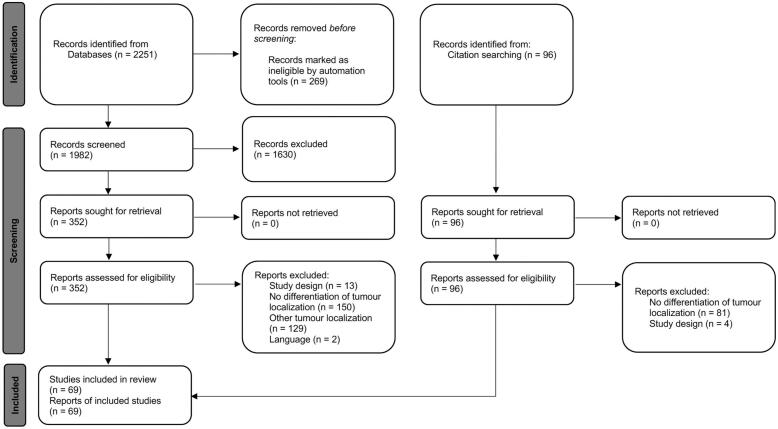
Literature search according to PRISMA-statement [15].

Trials reporting on *(1)* SBRT (defined as > 3 Gy per fraction using high-energy photons with curative intention) of *(2)* centrally or ultra-centrally located lung tumours and *(3)* outcome parameters (either overall survival (OS), progression-free survival (PFS), local control (LC) and toxicity) were included. Case reports, systematic reviews, comments, letters to the editor, non-English language articles and trials reporting on palliative treatment regimens or cohorts in which central and ultra-central tumour localization could not be clearly differentiated were excluded from analysis (Fig. 1).

### Data Collection

All manuscripts, supplementary data and trial protocols were screened and included in the analysis according to the inclusion criteria described above. The following data were collected: author, year of publication, study design, total number of patients, histology and size of tumour lesions, dose prescription, technique of applied radiotherapy, dose to organs at risk (OAR), definition of ultra-centrally located lung tumours and outcome parameters (acute and late toxicity, overall survival and progression-free survival).

Each dose prescription as reported in the original publication was converted to the biological effective dose (BED) assuming an ⍺/β-value of 10 for lung tumours and an ⍺/β-value of 3 for normal thoracic tissues using the following equation:

(1) $${\mathrm{BED}}_{\alpha /\beta }\;=\;nd\left(1+\frac{d}{\alpha /\beta }\right)$$

n = number of fractions

d = dose per fraction

Detailed information on actual delivered dose distributions or PTV coverage was not consistently available and thus not converted to the biological effective dose.

### Outcome definition

The primary outcome parameters were acute and late toxicity of SBRT in central and ultra-central lung tumours. Grading of toxicity events was based on Common Terminology Criteria for Adverse Events (CTCAE v5). Secondary outcome parameters were mean, 1- and 2- year OS, LC and PFS after completion of SBRT until last reported follow-up, respectively.

### Statistical analysis

Mean, median and standard error were calculated for all applicable data. Trials were omitted from analysis if certain of the above-mentioned endpoints were not available. While all eligible studies were included in the systematic review, only those providing extractable toxicity data were included in the quantitative *meta*-analysis.

Random-effect models with Freeman-Tukey double arcsine transformation were used to estimate the pooled effect size and its 95% confidential interval of observed studies [16], [17], [18]. *I^2^* was calculated to describe the heterogeneity with values of *I^2^* = 25% as low, *I^2^* = 50% as moderate and *I^2^* = 75% as high heterogeneity [19]. A p-value threshold of < 0.05 was determined as statistically significant.

Pearson’s *r* was used for correlation analysis between toxicity and radiation dose.

Data were managed by using Microsoft Excel version *16.84* (Microsoft, Redmond, WA, USA) and analysis was performed in R version *4.3.2* (R Foundation for Statistical Computing, Vienna, Austria), using the *meta* and *metafor* packages. Additional figures were plotted with GraphPad Prism version 10.6.1 (GraphPad Software Inc.).

## Results

### Characteristics of included trials

**Fig. 2:**
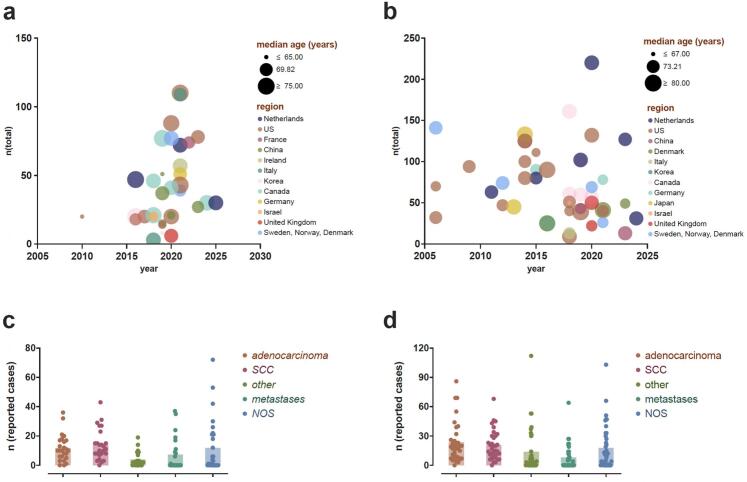
**A− d:** Cohort details in trials reporting on ultra-centrally located lesions [a] and centrally located lesions [b]. Mean of histological subgroups in reported ultra-centrally located lesions [c] and centrally located lesions [d].

69 of 2,251 screened studies, published between 2006 and 2025 met the inclusion criteria and were included in the final analysis. Most included studies had a retrospective study design, except 12 trials that were of prospective nature [9], [11], [12], [20], [21], [22], [23], [24], [25], [26], [27], [28]. The exact definition of ultra-central tumour localization varied between different trials, but tumours abutting the PBT were always defined as ultra-centrally located (37/37). In addition, ultra-central localization was reported as an overlap of the planning target volume (PTV) and the trachea in 32.4% (12/37), the vessels in 35.1% (13/37), the pericardial pleura in 8.1% (3/37), the heart in 8.1% (3/37) and the oesophagus in 45.9% (17/37). The mean planning target volume (PTV) was 50.1 (±25.4) cm^3^ for centrally and 59.9 (±26.2) cm^3^ for ultra-centrally located lesions. All characteristics of reported patient cohorts, tumour lesions, and different treatment protocols are summarized in Fig. 2 and Table 1.

**Table 1:** Prescription parameters and definition of ultra-centrally located tumours.

**author**	*PTV (ccm)*	*dose per fraction (Gy)*	*total dose (Gy)*	*BED3mean (Gy)*	*BED10mean (Gy)*	*dose prescription*	*PTV underdosage allowed*	*technique*	*definition of ultracentral lung-tumour*
Lodeweges et al. [29]	55,6	5	60	160	90	D95% > 100%; D99% > 90%; Dmax 145%	*yes*	IMRT; VMAT	PTV abutting or overlapping the main bronchi, trachea and/ or esophagus
Breen et al.[76]	56,5	7,5–12	48–60	185	101,7	*NA*	*NA*	VMAT	PTV overlapping PBT or GTV within 1 cm around PBT
Guillaume et al. [69]	51,6	5–10	34,2–60	*NA*	83,67	IDP 80%	*yes*	*NA*	PTV overlapped the trachea, right and left main bronchi, intermediate bronchus, lobe bronchi, oesophagus, heart
Wang et al. [47]	103	4–10	50–60	197,3	96,9	*NA*	*yes*	IMRT; VMAT	GTV overlapping the PBT or PTV overlapping the esophagus
Cong et al. (2)[62]	111,3	5	35	93,3	52,5	D95% > 100%; IDP 68–75%	*NA*	*NA*	GTV overlapping PBT or trachea
Rock et al.[30]	66,6	4–7	42,5–70	265,4	98,1	D95% > 100%	*yes*	IMRT; VMAT; 3DCRT	PTV overlapping or abutting the major vessels, esophagus or central airway
Mihai et al. [42]	57,84	5–12	40–60	210,8	102,9	IDP 98%	*yes*	IMRT	GTV abutting PBT or trachea
Sood et al. [31]	49,1	4–7	40–70	259,5	107,25	D95% > 100%	*NA*	*NA*	PTV in the mediastinum and hilum with directly abutting main airways, great vessels or esophagus
Loi et al.[48]	*NA*	5–10	48–70	*NA*	105	V95% > 95%, V98% > 98%, D2% < 107%	*yes*	VMAT	PTV overlapping PBT, esophagus, pulmonary vein or pulmonary artery
Yang et al. [33]	36,5	7,5	60	210	105	D95% > 100%	*NA*	VMAT	PTV abutting or overlapping PBT, heart and great vessels
Park et al. [77]	13,4	5–6	50–60	150,4	90,1	Dmax < 110%	*NA*	*NA*	PTV abutting PBT
Cong et al. (1)[78]	*NA*	5,2–6	31–36	NA	58,3	*NA*	*NA*	Cyberknife	GTV overlapping PBT or trachea
Repka et al.[41]	79,6	5–9	25–45	120,6	60,9	*NA*	*NA*	Cyberknife	PTV within lung parenchyma, hilum, or mediastinum with direct abutment of the trachea, mainstem bronchus, or esophagus
Tekatli et al.[14]	104,5	5	60	160	90	D95% > 100%; D99% > 90%, Dmax < 140%	*yes*	VMAT	PTV overlapping PBT or trachea
Unger et al.[32]	73	6–8	30–40	*NA*	*NA*	D95% > 100%	*NA*	Cyberknife	PTV overlapping PBT
Giuliani et al.[27]	*NA*	7,5	60	210	105	D95% > 100%; D99% > 90%, Dmax < 120%	*yes*	IMRT; VMAT; 3DCRT; Cyberknife	PTV overlapping central bronchial tree, esophagus, pulmonary vein or pulmonary artery
Wu et al. [26]	54,9	4–7.5	60	193,7	100,1	D95% > 100%; D99% > 90%; IDP 60–90%	*yes*	VMAT	PTV directly overlapping the proximal bronchial tree (PBT)
Nguyen et al.[34]	*NA*	7–10	40–60	204,7	97,33	D95% > 100%; D99% > 90%	*NA*	*NA*	PTV overlapping PBT or esophagus
Chang et al.[49]	78,9	6–10	30–50	*NA*	*NA*	V95% > 99%; Dmax < 120%	*NA*	IMRT; VMAT	ITV overlapping PBT
Lindberg et al.[25]	40,3	7	56	205,3	100,8	D95% > 95%; IDP 67%	*NA*	IMRT; VMAT; static-field	PTV in 1 cm range of PBT
Regnery et al.[50]	*NA*	5	50	133,3	75	IDP 95%	*yes*	VMAT; 3D; Tomotherapy	PTV overlapping PBT
Farrugia et al.[40]	33,6	10–12	50–60	185,3	107,6	D95% > 100%	*NA*	VMAT; 3DCRT	GTV abutting PBT, trachea, mediastinum, aorta or spinal cord
Chaudhuri et al.[13]	39,6	10–12,5	50	240,4	107,1	D95% > 100%; Dmax < 120%	*NA*	IMRT	GTV abutting PBT or trachea
Song et al.[35]	103	6–8	56–60	188,4	97,6	D95% > 100%; D99% > 90%; Dmax < 110%	*NA*	IMRT	PTV overlapping PBT, esophagus or vessels
Zhao et al.[36]	42,1	7,5	60	210	105	IDP 90%; V100% > 95%; V90% > 99%	*yes*	IMRT; VMAT; 3DCRT	PTV overlapping the PBT, esophagus or vessels
Lenglet et al.[51]	31,1	7–20	40–60	231,4	105,8	IDP 65–85%; Dmax < 120%	*yes*	VMAT; Cyberknife	PTV overlapping PBT, esophagus, great vessels and pericardial pleura
Raman et al.[52]	68,5	5–12	48–60	207,3	105,2	D99%> 90%; IDP 60–90%	*NA*	IMRT; VMAT	PTV abutting or overlapping the proximal PBT, trachea, esophagus or vessels
Meng et al.[53]	55	6–15	48–60	201	98,2	IDP 60–80%	*NA*	*NA*	PTV abutting PBT
Tonneau et al. [54]	*NA*	*NA*	*NA*	*NA*	*NA*	IDP 70–90%	*NA*	VMAT; Cyberknife; Tomotherapy	PTV overlapping PBT, esophagus, pulmonary vein or pulmonary artery
Karasawa et al.[43]	*NA*	3	75	150	97,5	*NA*	*NA*	*NA*	CTV faced the lobar bronchus, esophagus, or major vessels or PTV overlapping the trachea or main bronchus
Korzets et al.[55]	*NA*	7,5–18	48–60	*NA*	*NA*	D95% > 95%	*yes*	IMRT; VMAT	GTV with direct contact with the primary bronchial tree or espohagus
Haseltine et al.[46]	*NA*	9–10	45–50	188,2	88,7	IDP 100%	*NA*	IMRT	GTV touch or invade the trachea, a main stem bronchus, or a lobar bronchus
Cooke et al.[37]	32,8	7,5	60	210	105	D95% > 100%; Dmax < 130%	*yes*	IMRT; VMAT	GTV abutting PBT
Cho et al.[56]	*NA*	3	60	120	78	D95% > 97%	*NA*	*NA*	CTV is < 1,5cm distant to esophagus
Steber et al.[57]	*NA*	5–10	50	*NA*	*NA*	*NA*	*NA*	3D; IMRT; VMAT	PTV overlapping the proximal bronchial tree, esophagus, pulmonary vein, or pulmonary artery
Sahin et al.[38]	*NA*	*NA*	*NA*	*NA*	151,2	D95% > 100%	*NA*	*NA*	PTV < 1 cm from mediastinal structures
Figlia et al.[39]	*NA*	4	40	93,3	56	IDP 95%	*NA*	Tomotherapy	*NA*

NA = not available,

IDP = isodose .

### Ultra-centrally located tumours

#### Dose prescription and target volume definition

The dose prescription varied between studies. The mean BED_10_ was 94.5 (±19.1) Gy and the mean BED_3_ was 185.2 (±44.6) Gy with a most common prescription of D95% > 100% [13], [14], [26], [27], [29], [30], [31], [32], [33], [34], [35], [36], [37], [38], [39], [40]. In the majority of the observed studies, 4D-CT was used for an ITV-based treatment-planning (83.8% of 37 trials). Only two trials conducted deep inspiration-breath hold for radiation planning [41], [42]. PTV margins in all directions ranged between 3 – 10 mm (median 5 mm). Constraints of OARs varied between studies, while 37.8% (14/37) of all studies investigating ultra-centrally tumours prioritized OAR constraints over PTV coverage. Further details on chosen dose prescriptions are summarized in Table 1.

##### Outcome

The median overall follow-up was 21.2 (range 8.6 – 93.6) months. Large differences in overall survival (median 28.0; range 12.0 – 64.5 months) and progression-free-survival (median 12.0; range 7.0 – 36.0 months) were observed. Median 1- and 2-year overall survival were 78.6 (range: 45.0 – 92.7) % and 57.0 (range: 27.0 – 80.0) %, respectively (Table 2). Median 1- and 2-year local control were 87.9 (range: 30.0 – 98.0) % and 88 (range: 73.0 – 100.0) %.

**Table 2:** Outcome reported in included studies (ultra-centrally located lesions).

**author**	**year**	*n(total)*	*median follow-up*	*mean OS (years)*	*1-year OS (%)*	*2-year OS (%)*
Lodeweges et al. [29]	2021	72	19	29	77	52
Breen et al.[76]	2021	110	30	*NA*	78	57
Guillaume et al. [69]	2022	74	25	31	86,2	61,2
Wang et al. [47]	2020	88	19,6	38,6	78,6	64,5
Cong et al. (2)[62]	2019	51	17	18	76,5	38,9
Rock et al.[30]	2023	78	13,1	*NA*	89,2	*NA*
Mihai et al. [42]	2021	57	26,5	34,3	*NA*	55,1
Sood et al. [31]	2020	20	17,8	17,3	68	*NA*
Loi et al.[48]	2021	109	17	27	88	55
Yang et al. [33]	2020	21	15	15	87,5	76,6
Park et al. [77]	2019	8	8,6	*NA*	87,5	*NA*
Cong et al. (1)[78]	2019	15	16,5	17	66,7	27
Repka et al.[41]	2017	20	12	12	45	*NA*
Tekatli et al.[14]	2016	47	29,3	15,9	61,5	28,7
Unger et al.[32]	2010	20	10	*NA*	54	*NA*
Giuliani et al.[27]	2024	30	36	*NA*	*NA*	*NA*
Wu et al. [26]	2025	30	34,8	*NA*	90	70
Nguyen et al.[34]	2019	14	16,2	*NA*	*NA*	76
Chang et al.[49]	2018	46	14	24,5	*NA*	50,4
Lindberg et al.[25]	2021	39	24	*NA*	69	30,7
Regnery et al.[50]	2021	51	not reached	*NA*	*81,2*	54,9
Farrugia et al.[40]	2021	43	23,7	*NA*	*NA*	53,3
Chaudhuri et al.[13]	2015	7	21	*NA*	*NA*	80
Song et al.[35]	2023	27	41	48	*NA*	*NA*
Zhao et al.[36]	2020	41	22,9	55,6	92,7	79,8
Lenglet et al.[51]	2019	77	36	37	*NA*	*NA*
Raman et al.[52]	2018	21	21,4	23,8	*NA*	*NA*
Meng et al.[53]	2019	37	44,47	64,47	*NA*	*NA*
Tonneau et al. [54]	2023	65	37,6	37,3	*NA*	*NA*
Karasawa et al.[43]	2018	21	93,6	*NA*	*NA*	*NA*
Korzets et al.[55]	2018	20	18,3	*NA*	*NA*	*NA*
Haseltine et al.[46]	2016	18	22,7	*NA*	*NA*	63,9
Cooke et al.[37]	2020	6	11,6	*NA*	82,7	69,5
Cho et al.[56]	2016	20	12,6	*NA*	*NA*	74,4
Steber et al.[57]	2021	26	40,2	*NA*	*NA*	*NA*
Sahin et al.[38]	2020	77	27	*NA*	*NA*	*NA*
Figlia et al.[39]	2018	3	13	*NA*	*NA*	*NA*

median follow-up shown in months, OS = overall survival, NA = not available.

### Centrally located tumours

#### Dose prescription and target volume definition

For centrally located lesions, the mean BED_10_ was 102.7 (±15.4) Gy, the mean BED_3_ was 214.1 (±47.4) Gy and thus higher than the respective doses used for ultra-centrally located lesions. The most frequently selected dose prescriptions were similar to these in ultra-centrally tumours with D95% > 100%.

##### Outcome

The median overall follow-up was 22.7 (range: 10.6 – 93.6) months. With a median 1- and 2-year OS of 80.9 (range: 69.2 – 92.7) % and 63.9 (range: 43.0 – 86.2) %, OS was marginally higher than in patients with ultra-centrally located lesions (median 39.4; range 19.0 – 57.0 months) (Table 3). Median 1- and 2-year local control were 92 (range: 78.0 – 100.0) % and 85.2 (range: 67.0 – 96.7.0) %, respectively.

**Table 3:** Outcome reported in included studies (centrally located lesions).

**author**	**year**	*n(total)*	*median follow-up*	*mean OS (years)*	*1-year OS (%)*	*2-year OS (%)*
Baumann et al.[79]	2006	141	33	*NA*	84,4	*NA*
Bezjak et al.[11]	2019	100	37,1	39,9	*NA*	*NA*
Chang et al. (1)[80]	2008	27	17	*NA*	*NA*	*NA*
Chang et al. (2)[81]	2014	100	30,6	55,6	*NA*	*NA*
Davis et al.[82]	2015	111	17	24,8	89	72
Dujim et al. (1)[83]	2020	220	*NA*	*NA*	*NA*	55
Eriguchi et al.[84]	2016	25	25	*NA*	*NA*	61,8
Haasbeek et al.[44]	2011	63	35	47	85,7	69
Horne et al. [85]	2018	40	16,4	22,7	69,2	49,4
Joyner et al.[86]	2006	9	10,6	*NA*	*NA*	*NA*
Karlsson et al.[87]	2012	74	18,6	*NA*	*NA*	*NA*
Khalil et al. [88]	2021	41	18	34	*NA*	*NA*
Knap et al. [89]	2023	49	44	51	88	*NA*
Le et al.[20]	2006	32	18	*NA*	80,5	*NA*
Levy et al.[12]	2024	31	43,2	46	*NA*	*NA*
Manyam et al.[90]	2020	132	26	*NA*	*NA*	*NA*
Modh et al.[91]	2014	125	17,4	29,1	83	64
Nantavithya et al.[21]	2018	9	27	28	*NA*	*NA*
Nishimura et al. [92]	2014	133	33	*NA*	*NA*	*NA*
Roach et al.[22]	2018	51	17	19	*NA*	43
Rowe et al. [93]	2012	47	11,3	*NA*	*NA*	*NA*
Rulach et al.[23]	2020	50	25,2	27	80,1	67,6
Schanne et al.[94]	2015	90	18,8	21	72	*NA*
Stam et al.[95]	2019	102	34,8	42,7	78,7	55,4
Stephans et al.[96]	2009	94	15,3	*NA*	80,9	*NA*
Swaminath et al. [28]	2024	64	36,1	*NA*	*NA*	*NA*
Takahashi et al.[97]	2013	45	21,2	*NA*	70,4	62,1
Tekatli et al. (1) [45]	2015	80	45	38	81	62
Tekatli et al. (2) [24]	2023	127	40,5	25	*NA*	*NA*
Timmermann et al. [9]	2006	70	17,5	32,6	*NA*	54,7
Videtic et al.[98]	2014	80	18,4	*NA*	69,5	*NA*
Wu et al. [99]	2014	125	14,3	*NA*	*NA*	*NA*
Nguyen et al.[34]	2019	39	15,3	*NA*	*NA*	73
Chang et al.[49]	2018	61	14	26,6	*NA*	57,7
Lindberg et al.[25]	2021	26	24	*NA*	92	76,9
Regnery et al.[50]	2021	78	not reached	*NA*	*79,2*	55,4
Farrugia et al.[40]	2021	40	38,8	*NA*	*NA*	78,9
Chaudhuri et al.[13]	2015	27	18,5	*NA*	*NA*	63,2
Song et al.[35]	2023	13	41	not reached	*NA*	*NA*
Zhao et al.[36]	2020	57	22,9	55,6	92,7	79,8
Lenglet et al.[51]	2019	60	36	57	*NA*	*NA*
Raman et al.[52]	2018	161	21,4	39,4	*NA*	*NA*
Meng et al.[53]	2019	43	44,47	not reached	*NA*	*NA*
Tonneau et al. [54]	2023	229	37,6	48,5	*NA*	*NA*
Karasawa et al.[43]	2018	19	93,6	*NA*	*NA*	*NA*
Korzets et al.[55]	2018	50	18,3	55,2	*NA*	*NA*
Haseltine et al.[46]	2016	90	22,7	*NA*	*NA*	63,9
Cooke et al.[37]	2020	22	11,6	*NA*	82,7	69,5
Cho et al.[56]	2016	25	14,3	*NA*	*NA*	86,2
Steber et al.[57]	2021	63	40,2	*NA*	*NA*	*NA*
Sahin et al.[38]	2020	69	27	*NA*	*NA*	*NA*
Figlia et al.[39]	2018	13	13	*NA*	*NA*	*NA*

median follow-up shown in months, OS = overall survival, NA = not available.

### Toxicity

The reported toxicity was considerably low. In total, for ultra-centrally vs. centrally located lesions 41 vs. 42% grade 1, 37 vs. 34% grade 2, 13 vs. 17% grade 3, 1% grade 4 and 8 vs. 6% grade 5 toxicities were observed, respectively. A *meta*-analysis was conducted on cumulative grade 3–5 toxicity and grade 5 toxicity for both cohorts. There was a significant data gap regarding the differentiation between acute and late toxicity, so all reported toxicities were summarized in *meta*-analysis irrespective of the specific time point of first occurrence.

Four studies did not differ between toxicities in ultra-centrally and centrally located lesions and thus were excluded from the *meta*-analysis [25], [34], [38], [43].

Overall, lowest toxicity was observed with 60 Gy in 8 fractions for both ultra-centrally [26], [27], [33], [36], [37] and centrally located lesions [12], [28], [36], [44], [45].

#### Ultra-centrally located tumours

**Fig. 3:**
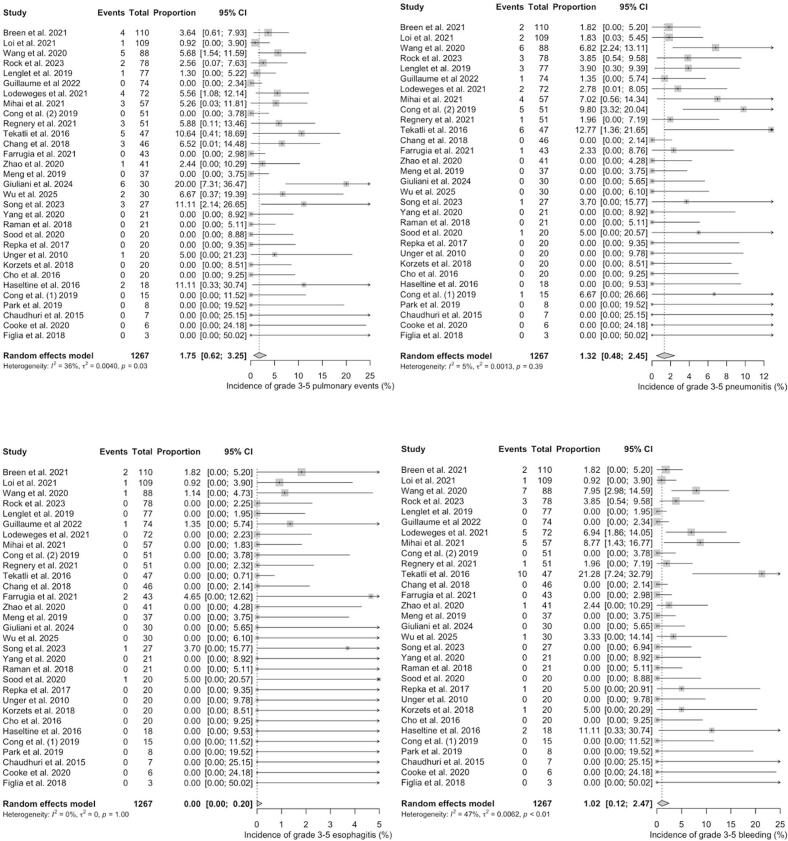
**A – d:** Meta-Analysis of Proportions of grade 3–5 toxicities in ultra-centrally located lesions (overall pulmonary toxicity [a], pneumonitis [b], esophagitis [c] and haemorrhage [d]). For Heterogeneity I^2^ was calculated.

**Fig. 4:**
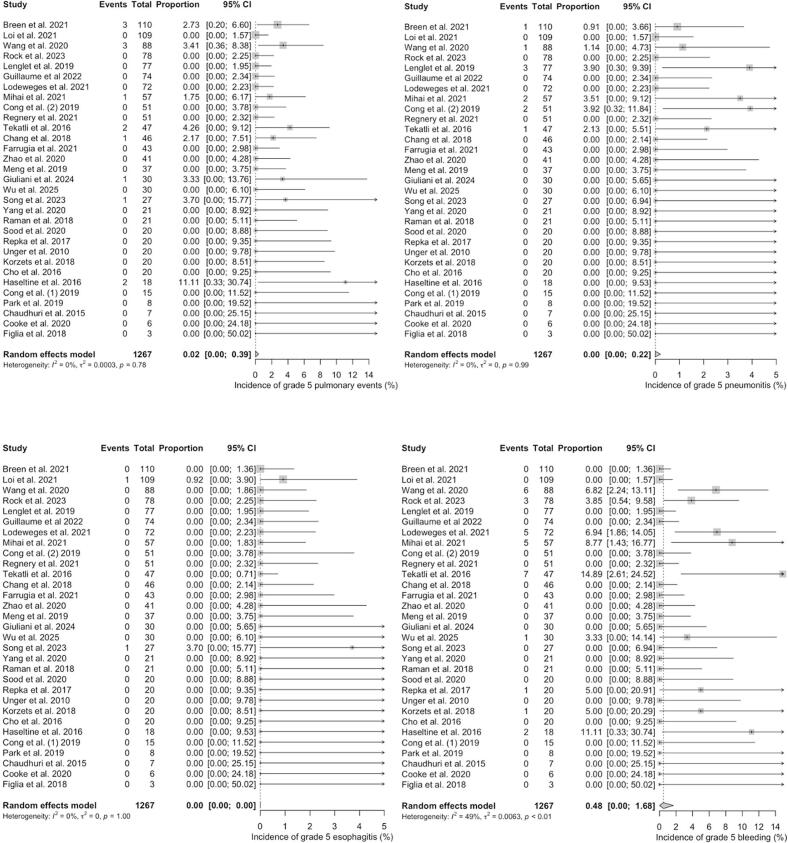
**A – d:** Meta-Analysis of Proportions of grade 5 toxicities in ultra-centrally located lesions (overall pulmonary toxicity [a], pneumonitis [b], esophagitis [c] and haemorrhage [d]). For Heterogeneity I^2^ was calculated.

For ultra-centrally located tumours, the pooled estimated proportions of grade 3–5 toxicities were 1.75% [range: 0 – 20; CI 95% = 0.62 – 3.25] for general pulmonary events (e.g. cough, dyspnea, respiratory failure, bronchial fistula, bronchial stenosis), 1.32% [range: 0 – 12.77; CI 95% = 0.48 – 2.45] for pneumonitis, 0.00% [range: 0 – 5; CI 95% = 0.00 – 0.20] for esophagitis and 1.02% [range: 0 – 21.28; CI 95% = 0.12 – 2.47] for bleeding, respectively, with low heterogeneity between the studies (*I^2^* = 36%, p = 0.03; *I^2^* = 5%, p < 0.39; *I^2^* = 0%, p = 1.00 and *I^2^* = 47%, p < 0.01) (Fig. 3*).* The most common reported pulmonary event was radiation pneumonitis (45.9% of all pulmonary events). For grade 5 toxicity alone, pooled estimated proportions were 0.02% [range: 0 – 11.11; CI 95% = 0.00 – 0.39] for pulmonary events, 0.00% [range: 0 – 3.92; CI 95% = 0.00 – 0.22] for pneumonitis, 0.00% [range: 0 – 3.70; CI 95% = 0.00 – 0.00] for esophagitis and 0.48% [range: 0 – 14.89; CI 95% = 0.00 – 1.68] for bleeding. Heterogeneity was low for studies reporting on esophagitis (*I^2^* = 0%, p = 1.00), pulmonary events (*I^2^* = 0%, p = 0.78) including radiation pneumonitis (*I^2^* = 0%, p = 0.99) and haemorrhage (*I^2^* = 49%, p < 0.01) (Fig. 4). The most frequently reported fatal (grade 5) complication was haemorrhage, observed in 31 out of 1,509 patients (2.1%). Herein, the correlation analysis failed to show any significant correlation between BED_3_ or BED_10_ and incidence of adverse events. A pooled quantitative risk estimation for specific risk factors was not feasible due to inconsistent reporting of statistical parameters (e.g. hazard or odds ratio) across studies. Nevertheless, several single studies reported potential risk factors for the occurrence of haemorrhage, including dose to the main bronchus such as D_mean_ < 91 Gy (BED_3_; OR 1.065) [29] and D_max_ < 119 Gy in EQD_2_ (α/β = 3) [25], the concurrent use of antiangiogenic drugs [46], [47] or previous interventions such as bronchial biopsy especially for tumours abutting bronchial structures [12].

Only two cases of grade 5 esophagitis were reported in two retrospective studies occuring with a D_max_ to the oesophagus of 51 Gy and 58.9 Gy (BED_3_). The corresponding mean BED_10_ were 105 Gy (for whole cohort with 48 – 70 Gy and 5 – 10 Gy/fx) [48] and 96 Gy (for the individual reported patient with 60 Gy and 6 Gy/fx) [35], respectively.

#### Centrally-located tumours

**Fig. 5:**
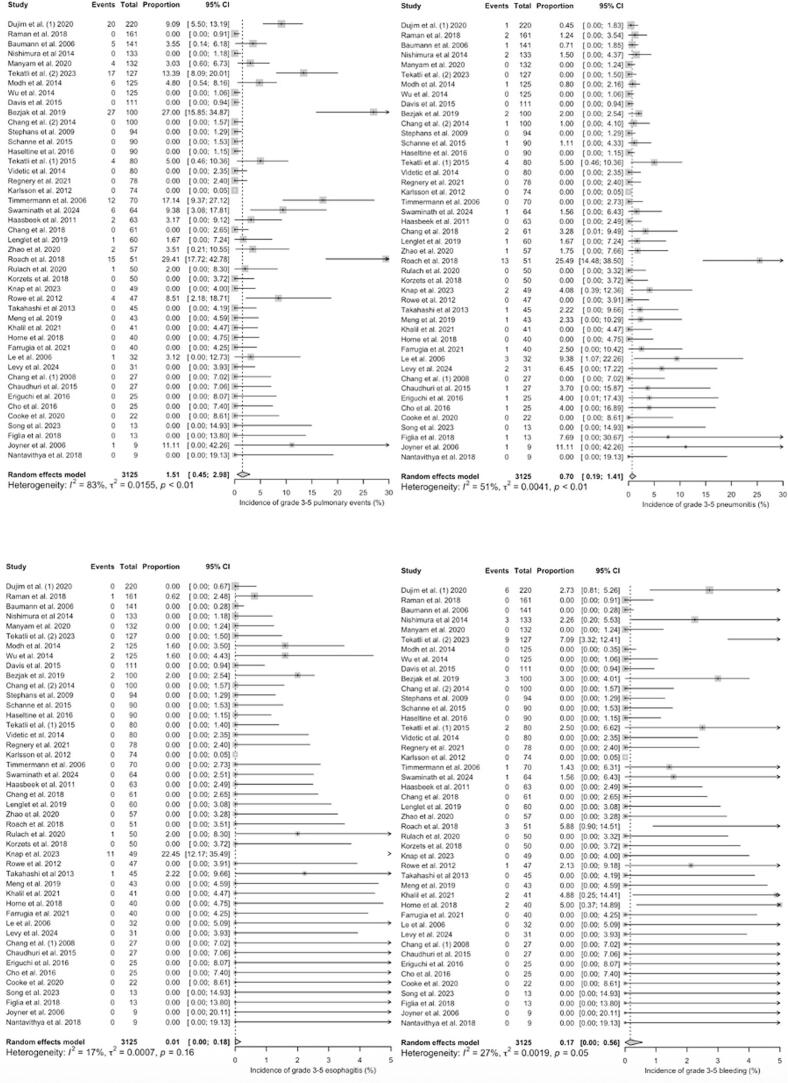
**A – d:** Meta-Analysis of Proportions of grade 3–5 toxicities in centrally located lesions (overall pulmonary toxicity [a], pneumonitis [b], esophagitis [c] and haemorrhage [d]). For Heterogeneity I^2^ was calculated.

In trials investigating centrally located tumours, pooled estimated proportions of grade 3–5 toxicities were 1.51% [range: 0 – 29.41; CI 95% = 0.45 – 2.98] for pulmonary events, 0.70% [range: 0 – 25.49; CI 95% = 0.19 – 1.41] for pneumonitis, 0.01% [range: 0 – 22.45; CI 95% = 0.00 – 0.18] for esophagitis and 0.17% [range: 0 – 7.09; CI 95% = 0.00 – 0.56] for bleeding. The heterogeneity between the trials was high for pulmonary events and moderate for pneumonitis (*I^2^* = 83%, p < 0.01; *I^2^* = 51%, p < 0.01) and low (*I^2^* = 17%, p = 0.16; *I^2^* = 27%, p = 0.05) for esophagitis and bleeding events (Fig. 5*).*

**Fig. 6:**
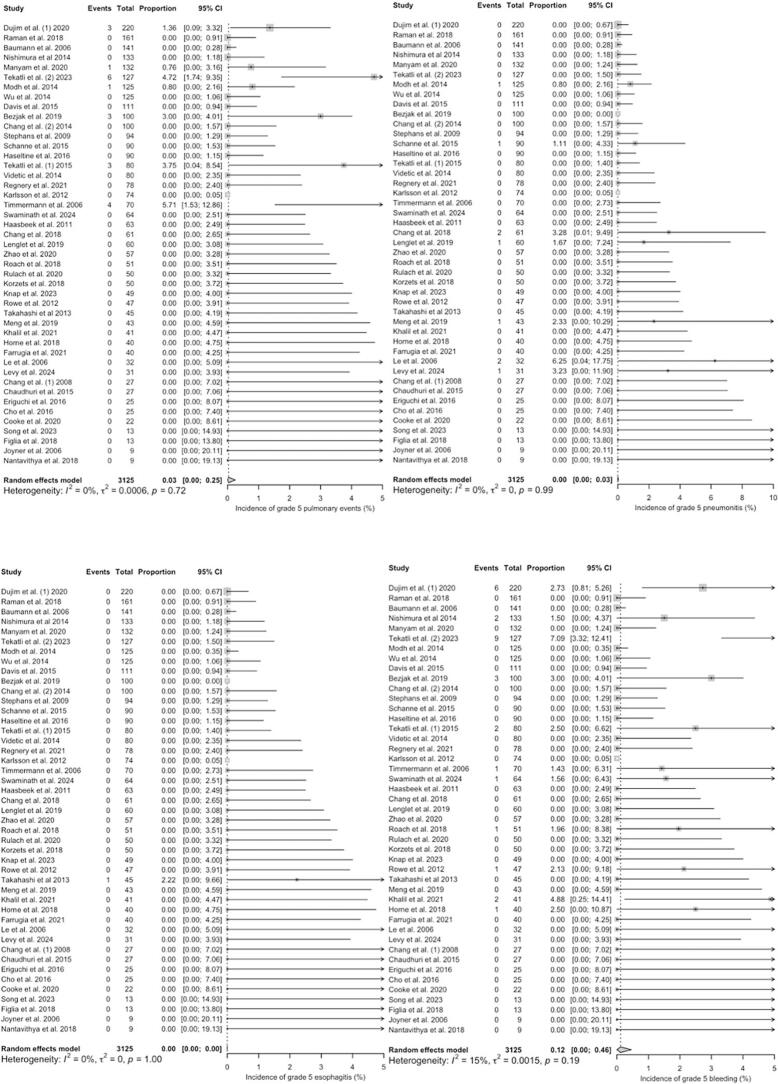
**A – d:** Meta-Analysis of Proportions of grade 5 toxicities in centrally located lesions (overall pulmonary toxicity [a], pneumonitis [b], esophagitis [c] and haemorrhage [d]). For Heterogeneity I^2^ was calculated.

Grade 5 toxicities were rarely reported with pooled estimated proportions of 0.03% [range: 0 – 5.71; CI 95% = 0.00 – 0.25] for pulmonary events, 0.00% [range: 0 – 6.25; CI 95% = 0.00 – 0.03] for pneumonitis, 0.00% [range: 0 – 2.22; CI 95% = 0.00 – 0.00] for esophagitis and 0.12% [range: 0 – 7.09; CI 95% = 0.00 – 0.46] for bleeding complications, respectively. The heterogeneity between the studies was low for all reported grade 5 events in centrally located tumours (Fig. 6).

### Subgroup analysis

#### Outcome

Twenty of all above included studies directly reported outcome data separately for centrally (n = 1185) and ultra-centrally (n = 659) located tumours [13], [25], [34], [35], [36], [37], [38], [39], [40], [43], [46], [49], [50], [51], [52], [53], [54], [55], [56], [57]. Within these trials, the reported median overall survival for centrally and ultra-centrally located lung tumours were 57.0 vs. 42.5 months and the median 2-year overall-survival rates were 73.0 vs. 78.0%, respectively.

#### Toxicity

Sixteen trials included in the previously conducted *meta*-analysis reported toxicities separately for centrally and ultra-centrally located lesions. Random-effects models were applied to estimate pooled proportions for each subgroup. No statistically significant differences in estimated pooled proportions of grade 3–5 toxicities between both subgroups were observed (p > 0.20; Supplementary Data 1) [13], [35], [36], [37], [39], [40], [46], [49], [50], [51], [52], [53], [54], [55], [56], [57]. Radiation pneumonitis occurred more frequently following SBRT of centrally located tumours with an estimated proportion of 0.71% [range: 0.00 – 7.69; CI 95% = 0.09 – 1.72] compared to 0.13% [range: 0 – 3.90; CI 95% = 0.00 – 1.28] in ultra-centrally located lesions. Interestingly neither bleeding complications nor esophagitis were reported more often in either subgroup within these trials (Supplementary Data 1). However, in univariate analysis of the different trials, ultra-central tumour location was often mentioned as a risk factor for haemorrhage. Lindberg et al. reported proportionally more common fatal bleeding events in ultracentral localization. Mean BED_3_ and BED_10_ were 205.6 and 100.8 Gy with a total dose of 56 Gy in 8 fractions. The reported D_max_ to vessels was 67 Gy and the D_max_ to the PBT was 119 Gy (EQD_2_ (α/β = 3)). Tumours located < 5 mm around the PBT had a significantly higher risk for the development of a fatal bleeding compared to tumours located 10 mm around the PBT (p < 0.05). [25].

## Discussion

To the best of our knowledge, this is the first comprehensive *meta*-analysis that systematically compares the efficacy and safety of SBRT for lung tumours according to tumour location. Despite high dose treatments (42 – 60 Gy total dose with 5 – 20 Gy per fraction), the overall pooled estimated proportions of fatal toxicities were low, with the highest risk observed for bleeding complications in 2.1% (for ultra-centrally located tumours) and 0.78% (for centrally located tumours) of patients. Interestingly, for both cohorts incidence of haemorrhage was associated with lethal complications and 77.5% (ultra-central tumours) vs. 87.5% (central tumours) of patients, who suffered a haemorrhage, died. Other severe toxicities were rare.

In this report, 60 Gy in 8 fractions was associated with favourable local control rates for both ultra-centrally [26], [27], [33], [36], [37] and centrally located lesions [12], [36], [44], [45]. While achieving convincing tumour control, high grade toxicities were extremely rare, making it a promising concept to consider for SBRT of both centrally and ultra-centrally located lung tumours. This is in line with recommendations of existing prescription guidelines for SBRT of centrally located lung tumours, while in ultra-centrally located tumours lower doses per fraction are commonly recommended [58], [59].

Recently published results of the prospective SUNSET and OCOG LUSTRE trials were very promising in terms of safety and efficacy of SBRT with 60 Gy in 8 fractions (BED_10_ 105 Gy). In SUNSET only one of 30 patients with ultra-central lung lesions suffered grade 5 toxicities during follow up, while 3-year local control and overall survival were excellent (89.6% and 72.5%) [27]. 3-year local control and overall survival rate of 85% and 58% as well as low fatal toxicity rate (4.3% of 23 treated patients) were reported for SBRT with 60 Gy in 8 fractions in sub-analysis of patients with centrally located lung lesions (OCOG LUSTRE, [26]). These results are consistent with retrospective studies that chose concepts with a comparable BED_3_ and BED_10_ reporting a 2-year local control of 85.7–93.7% and 2-year overall survival of 69.5%-79.8% without any occurrence of grade 5 toxicity [33], [36], [37].

Overall outcome and toxicity were also in line with a previously conducted systematic review by Yan et al. encompassing 1183 ultra-centrally located lesions. Therein, LC rates were reported and pooled with notably high 1- and 2-year rates of 92% and 89%, respectively. Reported treatment related risks were 4% for overall grade 5 toxicities and with 1% for grade 5 haemoptysis. [60] Also Rim et al. reported excellent control rates of SBRT with a 2-year local control rate of 96.7% and 2-year overall survival rate of 57.7% [61].

However, interpretation of fractionation regimens must not consider only the absolute prescription dose. Evidence from prospective trials suggests that the prescription technique and dose heterogeneity within the PTV may substantially influence occurrence of toxicity. In subgroup analysis of OCOG-LUSTRE, severe toxicity was associated with higher volumetric doses to the proximal bronchial tree as well as Dmax > 130% within the PTV [26]. Similarly, in the HILUS-trial, where 56 Gy in 8 fractions were prescribed to the 67% isodose, maximum doses within the PTV exceed 150% of the prescribed dose [25]. These findings indicate that excessive dose inhomogeneity and high volumetric exposure of critical mediastinal structures may outweigh differences in the absolute values of the prescription alone. Therefore, careful consideration of dose heterogeneity and strict prioritization of organs-at-risk constraints are essential, when applying regimens such as 60 Gy in 8 fractions for central and ultra-central located lung tumours.

One additional key aspect of SBRT assessment is the careful consideration of risk factors linked to the occurrence of grade 5 toxicities. Especially an overlap between the PTV and the PBT as well as the concurrent administration of anti-angiogenic drugs were identified as factors associated with higher risk for grade 5 bleeding events in previous studies [14], [25], [29], [46], [47]. But also previous interventions in bronchial structure, pre-existing interstitial lung disease and larger PTV (>150 cc) were associated with the occurrence of grade 5 adverse events and therefore adjustments of fractionation schemes should be considered in patients with these risk factors [12], [14], [32], [62]. Notably, the ASPIRE-ILD trial demonstrated feasibility and efficacy of SBRT with 50 Gy in 5 fractions in patients with pre-existing interstitial lung disease. However, tumour localization was not explicitly specified in this trial and reported toxicity were higher than in our pooled analysis, with haemorrhagic events in 2.6% (grade 3–5) and fatal pulmonary events in 7.7% (grade 5) of all patients [63]. These findings suggest that patient-related vulnerability and OAR-dose exposure contributed substantially to the observed outcome. Consequently, comparisons of fractionation regimens should be interpreted within their specific clinical context rather than solely on the basis of the BED values.

In conclusion, the above-mentioned fractionation concept may provide a favourable balance between tumour control and toxicity and can therefore be recommended for treating central and ultra-central lung tumours at an early stage. As OAR constraints were prioritized over PTV coverage in most trials investigating SBRT with 60 Gy in 8 fractions, OAR dose recommendations should be strictly followed in clinical routines when using this fractionation concept. The most important OAR to consider are PBT, esophagus and large vessels. For these, previous studies recommend a Dmax < 42 Gy (EQD_2_ (α/β = 3) 69.3 Gy),<40 Gy (EQD_2_ (α/β = 3) 64 Gy) and < 45 Gy (EQD_2_ (α/β = 3) 77.6 Gy) [64], [65], [66], [67]. Considering the low toxicity observed across all examined studies, dose levels below 60 Gy, combined with constraints for PBT analogous to those applied in the LungTech Trial, seem feasible [68].

Furthermore, quality assurance (QA) processes are essential to ensure that dose constraints for OAR are identified, enabling the development of effective dose delivery concepts. This does not only enhances treatment efficacy but also reduces the likelihood of adverse events and improves patient outcomes. [12], [64].

Several limitations should be considered when interpreting the results of this analysis. First, definition of ultra-centrally located tumours varies significantly in the literature. Differences in anatomical criteria, such as proximity to the proximal bronchial tree, the trachea, the oesophagus or the major vessels introduce relevant heterogeneity and may have influenced the pooled estimated toxicity. This is especially important for rare but clinically significant fatal events, where even small discrepancies in the definition of lesional localization may substantially affect risk estimation. Furthermore, acute and late toxicities were pooled in the quantitative analysis due to incomplete reporting of toxicities across several studies. This methodological limitation may have led to an underestimation of late and potentially fatal complications, particularly vascular or pulmonary events.

Second, there is a large heterogeneity regarding treatment prescription as well as planning techniques and reported outcome [51], [53], [69]. The majority of data are derived from retrospective cohorts, which introduces inherent selection bias and variability in reporting standards. Additionally, dosimetric information and data on PTV coverage were inconsistently reported. Therefore, conclusions regarding the apparent favourable fractionation concept of 60 Gy in 8 fractions should be interpreted with caution. While this regimen appears reasonable based on the available pooled data, the reported occurences of fatal haemorrhage in ultra-centrally located lung tumours underline that there is no absolute guarantee regarding safety of SBRT in critical regions and prospective validation remains necessary.

Third, the absence of a significant correlation of BED and toxicity outcome should be interpreted carefully. Reporting of organ-at-risk doses, prescription isodose levels and detailed dosimetric parameters were heterogeneous and partially lacking across the included studies. Consequently, BED alone may not adequately reflect clinically relevant dose-toxicity relationships in ultra-centrally and centrally located lung tumours in this analysis.

Finally, the impact of tumour biology on treatment response and outcome remains unclear. Some previously published studies reported inferior response to SBRT for squamous cell carcinoma [70]. In this *meta*-analysis, the distribution of different histological entities was rather heterogeneous, and detailed information on tumour biology were widely lacking. In this context, it is important to comprehend the radiobiological mechanisms of haemorrhage and necrosis in order to find an optimal fractionation scheme tailored to the specific tumour localisation on the one hand and the patient's individual risk factors on the other.

Given these limitations and the limited comparability of cohorts and outcome parameters, further prospective trials are warranted to conclusively derive treatment recommendations for patients with early-stage, ultra-central lung tumours. In addition, advanced technologies including MR-guided radiotherapy and adaptive treatment strategies should be considered, regarding potential reduction of unintended organ-at-risk exposure by reducing margins, daily adaption of treatment plans or improved soft-tissue visualisation, Similarly, proton therapy may offer dosimetric advantages when tumours are located in close proximity to radiosensitive mediastinal structures.

Promising prospective trials concerning dose prescription and constraint recommendations, like STRICT-LUNG (NCT05354596) and LungSTAAR (NCT04917224) are still ongoing and final results are pending. The MAGELLAN-trial will investigate the benefit of MR-guidance to optimize SBRT planning [71]. These trials are expected to provide further insights into whether these technological advances can improve the therapeutic ratio in lung tumours located in critical anatomical regions.

In addition, the combination of immune- and radiotherapy remains a highly controversial topic in the treatment of ultra-central lung tumours, especially regarding distant control. Recently reported or ongoing trials such as *ISABR (*NCT03148327), *KEYNOTE-867* (NCT03924869) and *PACIFIC-4* (NCT03833154) are investigating the compatibility of immunotherapies (e.g. durvalumab or pembrolizumab) with different fractionation schemes of SBRT. While early-phase data from ISABR suggested encouraging tumour control with acceptable toxicity, *KEYNOTE-867* was stopped early due to increased toxicity and lack of efficacy for SBRT combined with pembrolizumab compared to SBRT alone [72], [73], [74]. The results of *PACIFIC-4* are still pending and will contribute to an enhanced understanding of optimized treatment strategies and prescription recommendations for early-stage lung cancers in general [75]. Importantly, the present analysis did not systematically assess the impact of concurrent immunotherapy on toxicity outcomes in centrally or ultra-centrally located lung tumours. Given the already elevated risk of severe and potentially fatal adverse events in these anatomical regions, a careful evaluation remains essential to discern whether novel combination therapies adversely affect the overall outcome in this particular subgroup.

## Conclusion

Stereotactic body radiation therapy (SBRT) represents a feasible treatment approach for patients with early stage centrally and ultra-centrally located lung tumours, achieving convincing local control but remains with a moderate but clinically relevant risk of toxicity. Therefore treatment should be planned and delivered with particular caution. 60 Gy in 8 fractions appears to be a promising fractionation scheme for tumours of both localizations. However, to mitigate the risk of severe and potentially fatal toxicities, strict adherence to organ-at-risk constraints and careful individualised treatment planning are essential.

## Declaration of Generative AI and Ai-Assisted Technologies in the Writing Process

*During the preparation of this work the authors used ChatGPT (GPT4o, OpenAI) in order to optimize phrasing. After using this tool, the authors reviewed and edited the content as needed and take full responsibility for the content of the published article.*

## CRediT authorship contribution statement

**Shari Wiegreffe:** Investigation, Formal analysis, Data curation, Visualization, Writing – original draft. **Sonja Adebahr:** Writing – review & editing. **Tanja Schimek-Jasch:** Writing – review & editing. **Julian Philipp Layer:** Writing – review & editing. **Cas Stefaan Dejonckheere:** Writing – review & editing. **Gustavo Renato Sarria:** Writing – review & editing. **Davide Scafa:** Writing – review & editing. **Youness Nour:** Writing – review & editing. **Lara Caglayan:** Writing – review & editing. **Dimos Baltas:** Writing – review & editing. **Christos Moustakis:** Writing – review & editing. **Nils Henrik Nicolay:** Writing – review & editing. **Andreas Rimner:** Writing – review & editing. **Anca-Ligia Grosu:** Writing – review & editing. **Ursula Nestle:** Writing – review & editing. **Eleni Gkika:** Conceptualization, Methodology, Supervision, Writing – review & editing.

## Funding

This research did not receive any specific grant from funding agencies in the public, commercial, or not-for-profit sectors.

## Declaration of competing interest

Andreas Rimner reports a relationship with AstraZeneca GmbH that includes: consulting or advisory, funding grants, and travel reimbursement. Andreas Rimner reports a relationship with Merck & Co Inc that includes: board membership, consulting or advisory, and funding grants. Andreas Rimner reports a relationship with MoreHealth that includes: consulting or advisory. Andreas Rimner reports a relationship with Boehringer Ingelheim GmbH that includes: speaking and lecture fees. Andreas Rimner reports a relationship with ITMIG that includes: board membership. Andreas Rimner reports a relationship with IMIG that includes: board membership. Andreas Rimner reports a relationship with ABR that includes: board membership. Sonja Adebahr reports a relationship with BMBF - Federal Ministry of Education and Research that includes: funding grants. If there are other authors, they declare that they have no known competing financial interests or personal relationships that could have appeared to influence the work reported in this paper.
