# Supplementary material for: Outcome and safety of SBRT in centrally and ultra-centrally located lung tumours: A PRISMA-based systematic review and Meta-Analysis
Source: Clin Transl Radiat Oncol. 2026 Mar 21;59:101151. doi: 10.1016/j.ctro.2026.101151 (PMC13053848; doi:10.1016/j.ctro.2026.101151)
Supplement: Supplementary Data 1 [file mmc1.docx]

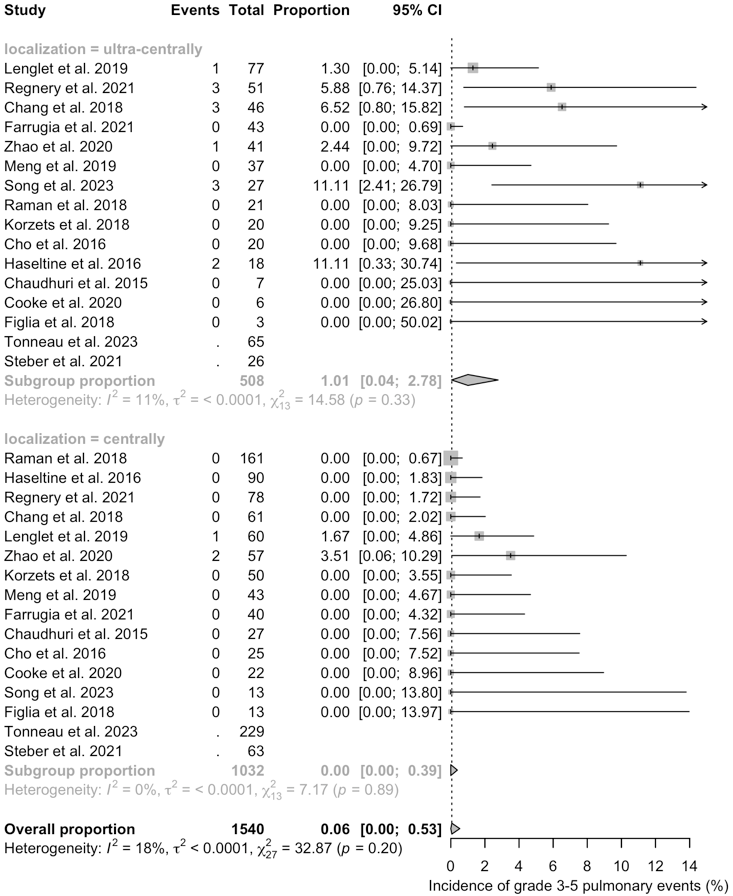

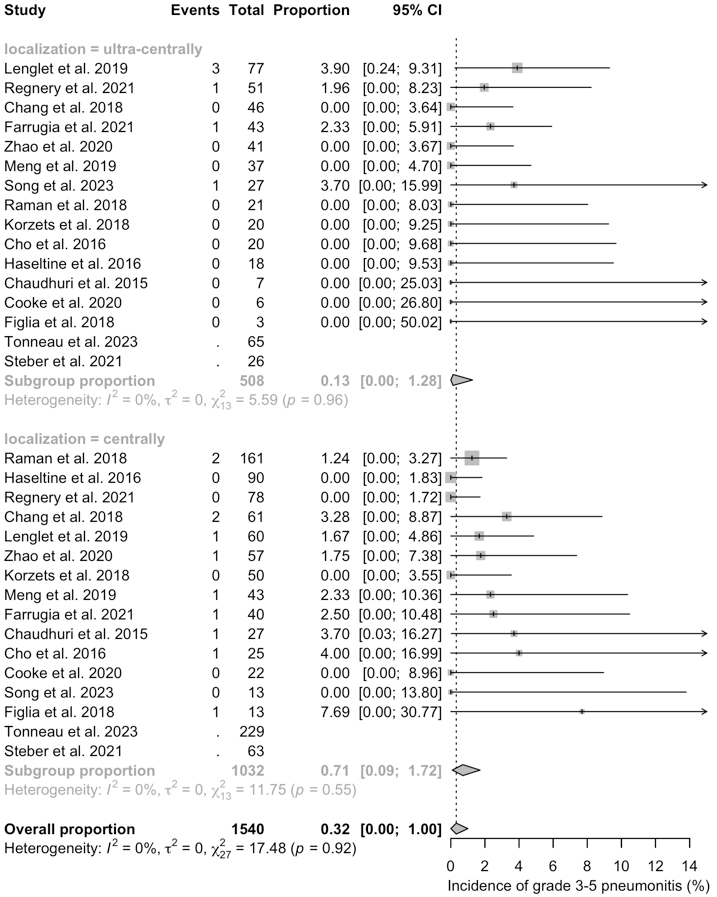


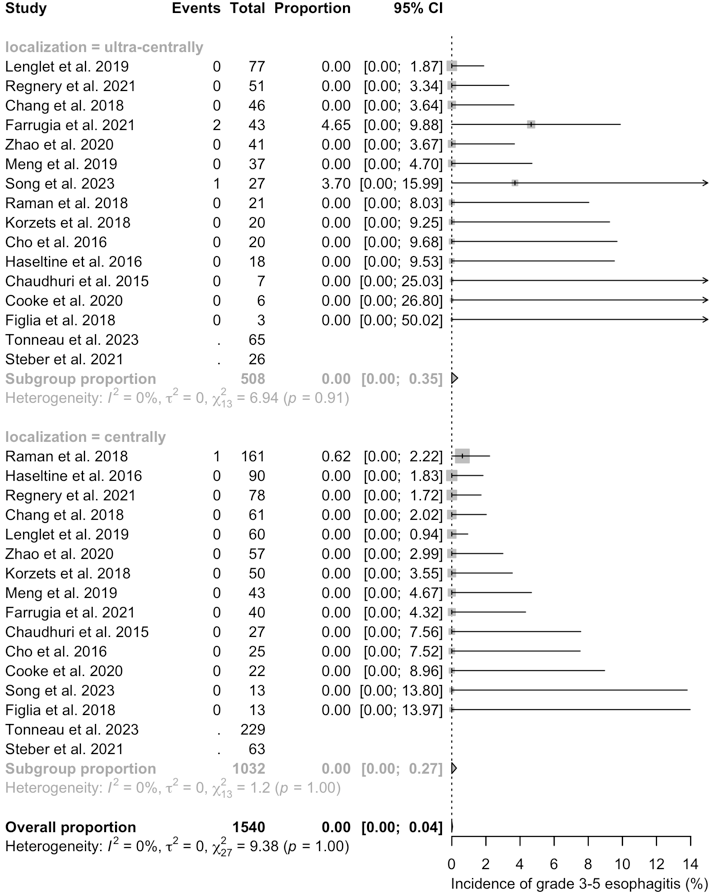

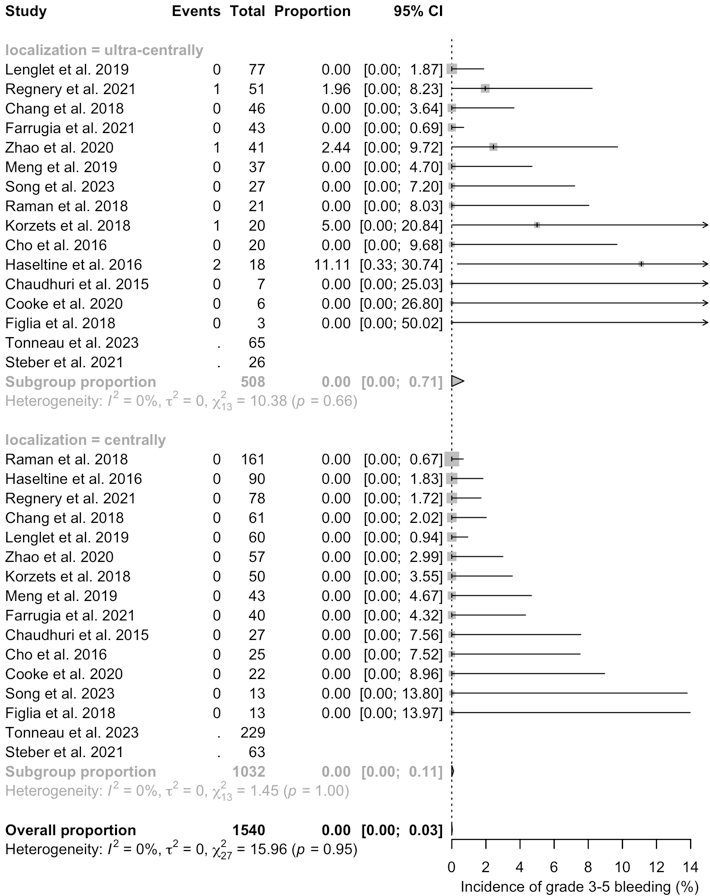


**Supplementary Figure 1a – d:** *Meta-Analysis of Proportions of grade 3-5 toxicities in ultra-centrally and centrally located lesions (overall pulmonary toxicity [a], pneumonitis [b], esophagitis [c] and haemorrhage [d]). For Heterogeneity I^2^ was calculated.*
